# Early Therapeutic Drug Monitoring for Isoniazid and Rifampin among Diabetics with Newly Diagnosed Tuberculosis in Virginia, USA

**DOI:** 10.1155/2013/129723

**Published:** 2013-11-17

**Authors:** Scott K. Heysell, Jane L. Moore, Debbie Staley, Denise Dodge, Eric R. Houpt

**Affiliations:** ^1^Division of Infectious Diseases and International Health, University of Virginia, P.O. Box 801340, Charlottesville, VA 22908-1340, USA; ^2^Tuberculosis Control and Prevention, Virginia Department of Health, Richmond, VA, USA

## Abstract

Slow responders to tuberculosis (TB) treatment in Virginia have prolonged treatment duration and consume more programmatic resources. Diabetes is an independent risk factor for slow response and low serum anti-TB drug concentrations. Thus, a statewide initiative of early therapeutic drug monitoring (TDM) for isoniazid and rifampin at 2 weeks after TB treatment was piloted for all diabetics with newly diagnosed TB. During the period of early TDM, 12/01/2011–12/31/2012, 21 diabetics had *C*_2 hr_ concentrations performed and 16 (76%) had a value below the expected range for isoniazid, rifampin, or both. Fifteen had follow-up concentrations after dose adjustment and 12 (80%) increased to within the expected range (including all for rifampin). Of 16 diabetic patients with pulmonary TB that had early TDM, 14 (88%) converted their sputum culture to negative in <2 months. Early TDM for diabetics was operationally feasible, may speed response to TB therapy, and can be considered for TB programs with high diabetes prevalence.

## 1. Introduction

Tuberculosis (TB) and diabetes mellitus have been described as the “convergence of two epidemics” and given the increasing rates of obesity and diabetes worldwide and the continued high rates of TB in low-income countries, it is estimated that the number of individuals with both TB and DM will increase dramatically [1]. Compared to those without diabetes, diabetics are at greater risk for incident TB [2, 3], where for instance, among Hispanic people aged 25–54 years, TB risk attributable to diabetes is estimated at 25% [1]. The largest meta-analysis to date demonstrated that diabetic patients were 3.1 times more likely to develop TB than nondiabetics, a risk which was amplified in regions outside North America [3]. 

Furthermore, when active TB disease develops, diabetes contributes to increased severity and poor treatment outcome [4]. Diabetics with TB appear more likely to die than non-diabetics with TB when adjusting for comorbid conditions and have higher rates of relapse after treatment completion [5, 6]. In Virginia, diabetics were 7 times more likely to have slow response to TB therapy [7]. In addition to other complications of treatment, slow response prolongs infectiousness and often extends the treatment duration. While other comorbid immunosuppressing conditions or *Mycobacterium tuberculosis* drug resistance certainly contribute to slow response in Virginia, uniquely the majority of diabetics had serum anti-TB drug concentrations of isoniazid and rifampin below the expected range, and diabetes was an independent risk factor for low rifampin concentrations [7]. We further demonstrated that patients with similarly low serum concentrations of isoniazid or rifampin have impaired killing of their own *M. tuberculosis* isolate in a functional bioassay [8]. Yet in an individual diabetic, low serum concentrations cannot be reliably predicted and may be a consequence of inaccurate dosing, drug solubility, malabsorption, altered metabolism or protein binding, or drug-drug interactions [9]. In most instances, however, low serum concentrations of isoniazid and rifampin can be readily corrected with dose adjustment. We previously observed that low rifampin concentrations that were then corrected by dose adjustment in Virginia were not only well tolerated, but also those patients had a shorter duration of TB therapy compared to other slow responders [7]. Aside from early diagnosis and treatment of unrecognized diabetes in TB patients, few other interventions to improve treatment outcome have focused on this vulnerable population.

The measurement of anti-TB drug concentrations, termed therapeutic drug monitoring (TDM), has been in use in Virginia since 2007 [10]. Statewide guidelines for TDM have been developed for patients slow to respond to TB treatment at 4–6 weeks of therapy, specifically if the patient has persistent or worsening TB symptoms or for pulmonary TB patients, a lack of decrement in the mycobacterial burden in the sputum [11]. Once slow response is identified, serum is collected for TDM to isoniazid and rifampin at the time of estimated peak concentration (Table 2), and a single dose adjustment is made if a concentration is below the minimum of the expected range (e.g., rifampin 600 mg daily increased to 900 mg daily). Since the routine use of TDM, diabetics accounted for 10–15% of the annual TB cases, but 40% of those with slow response [10, 12]. As a consequence, a recent statewide initiative was begun to perform TDM at 2 weeks following TB treatment initiation (early TDM) in all diabetics with newly diagnosed TB in an effort to correct low drug concentrations and prevent slow response. The following report describes the programmatic results of the initiative.

## 2. Methods

### 2.1. Study Design

A retrospective analysis was performed of all patients in whom TDM was completed during the period of the early TDM initiative, 12/01/2011 to 12/31/2012. Specifically, diabetics with early TDM at 2 weeks were compared to non-diabetics that had standard TDM for slow response. Surveillance data were retrieved from the state TB registry and included demographics (age, sex, and country of origin), comorbidities including HIV and diabetes, prior TB history, and anatomic focus of current TB episode (pulmonary, extrapulmonary, or both). Laboratory report forms were reviewed for drug concentration results for isoniazid and rifampin. Non-diabetic patients that had TDM for reasons other than slow response and those with TDM for second-line medications used in the treatment of drug-resistant TB were excluded. For diabetics with pulmonary TB, the time in days to sputum culture conversion was also assessed as a marker for prevention of slow response, given that the intensive phase of treatment and/or the total treatment duration were commonly extended for patients that fail to convert their sputum culture to negative in <2 months. Standard procedure was for sputum collection weekly until smear conversion and monthly until culture conversion. The study was given exempt approval by the Institutional Review Board at the University of Virginia and the Virginia Department of Health.

### 2.2. Early TDM Initiative

**Figure 1:**
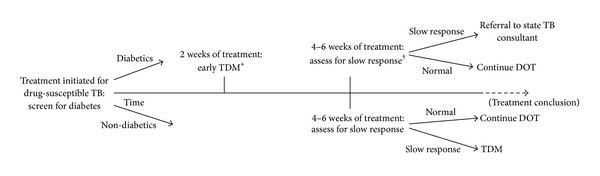
Statewide guidelines for the use of therapeutic drug monitoring (TDM). Diabetes identified by patient self-report or review or chart review by tuberculosis (TB) nurse case managers. *TDM: an estimated peak concentration (*C*_2 hr_) for isoniazid and rifampin is collected following directly observed therapy (DOT) and if below the expected range, then a single dose adjustment is made as per guidelines (e.g., rifampin 600 mg daily increased to 900 mg daily or isoniazid 300 mg daily increased to 450 mg daily) [11]. ^†^Slow response defined as persistent or worsening symptoms of TB or lack of decrement in mycobacterial burden in sputum for pulmonary TB patients [11].

In Virginia, all cases of active TB are reported to the State Department of Health, and each case is assigned to a nurse manager. Directly observed therapy is administered by the nurse case manager or a trained outreach worker. Diabetes is determined by self-report of the patient or caregiver to nurse case managers. During the early TDM initiative, nurse case managers also queried for use of insulin among diabetics. The use or type of oral hypoglycemic was not routinely recorded. Laboratory markers of disease severity such as glycosylated hemoglobin (HbA1c) were not available for analysis. Patients with diabetes had early TDM for isoniazid and rifampin at or as close to 2 weeks from treatment initiation for drug-susceptible TB, the earliest time point at which steady state concentrations are observed (Figure 1). The standard procedure for TDM was to directly administer medication and then collect venous blood 2 hours later at the time of estimated peak concentration (*C*_2 hr_) [11]. Serum was then separated by centrifugation at the Local Health Department before being transported on dry ice to the Regional Referral Laboratory. High performance liquid chromatography (HPLC) results were available within 48 hours of receipt of specimen and reported in reference to the expected *μ*g/mL range [9]. For concentrations of either isoniazid or rifampin below the *C*_2 hr_ expected range, a single dose increase was performed with plan to recheck the drug concentration following dose adjustment. For daily dosed rifampin of 600 mg, the dose was increased to 900 mg; for daily dosed isoniazid of 300 mg, the dose was increased to 450 mg. Intermittent dosing of rifampin was unchanged from daily dose adjustment, while isoniazid of 900 mg (typical intermittent dosing) was increased to 1200 mg. Providers were encouraged to initiate therapy with daily dosing for diabetic patients, but initial decisions were determined by the individual provider. 

Following TDM, patients were further monitored for slow response as defined by state guidelines [11] and if later identified, then referral was made to a state TB consultant (Figure 1). Patients without diabetes had TDM performed for isoniazid and rifampin only after the development of slow response. If drug concentrations remained below the expected range following dose adjustment in non-diabetic slow responders, then similar referral was made to a state TB consultant. Complications with dose adjustment or major toxicity were reported to the state TB control program.

### 2.3. Statistical Analysis

Demographic and clinical characteristics were compared between diabetics with early TDM and non-diabetics with standard TDM for slow response with the *χ* ^2^ statistic or for continuous variables, the Student *t*-test, or the Mann-Whitney *U* test when appropriate. *C*_2 hr_ values were dichotomized into “normal” if were within or above the expected range or “low” if were below the expected range. The biweekly or thrice weekly dosing used in some patients for isoniazid and rifampin was categorized as intermittent. Bivariate logistic regression analysis was used to determine additional risk factors for either a low isoniazid or a low rifampin concentration among diabetics. All tests of significance were two-sided.

## 3. Results

During the study period, 266 cases of active TB were on treatment. Seven (3%) patients were excluded from the analysis as TDM was performed for reasons other than diabetes or slow response, such as the use of second-line drugs in the treatment of drug-resistant TB or first-line drug intolerance. Twenty-one diabetics (81% of total eligible diabetics) had early TDM with complete lab report forms available for review, and 14 non-diabetics had standard TDM for slow response (Table 1). Diabetics were older with mean age of 57 ± 17 years compared to slow responders, 46 ± 12 years (*P* = 0.04). The majority of patients in whom TDM was performed were male, foreign born, and with pulmonary TB, but without significant difference in these proportions between diabetics and slow responders. Ten (48%) of diabetics were insulin dependent. No diabetic had HIV or a prior history of TB. The median time to TDM after treatment initiation in diabetics was 23.4 ± 16 days and expectedly differed from slow responders, 88 ± 54 days (*P* = 0.003).

Initial TDM for isoniazid was successfully performed in all patients, including 4 (19%) of diabetics and 6 (43%) of slow responders on intermittent dosing schedules; and for rifampin it was performed in 20 (95%) of diabetics and all slow responders. Mean *C*_2 hr_ values of daily dosed isoniazid were 2.13 ± 1.5 *μ*g/mL for diabetics compared to 3.1 ± 1.1 *μ*g/mL in slow responders (expected range 3–6 *μ*g/mL) (*P* = 0.11). While mean values for intermittent doses were 6.0 ± 3.0 *μ*g/mL for diabetics compared to 11.3 ± 2.5 *μ*g/mL in slow responders (expected range 9–18 *μ*g/mL) (*P* = 0.03). Fourteen (67%) of diabetics had a low isoniazid *C*_2 hr_ compared to 6 (50%) slow responders (*P* = 0.29). A low rifampin *C*_2 hr_ was observed in 12 (60%) diabetics, including 7 (70%) of insulin-dependent diabetics, compared to 5 (41%) of slow responders (*P* = 0.31). 

Overall, 16 (76%) of diabetics had a low isoniazid or rifampin *C*_2 hr_. Insulin use was not additionally predictive of a low concentration of either medication among diabetics in bivariate analysis; odds ratio were 2.3 (0.37–14.6) (*P* = 0.37) for rifampin and 1.3 (0.21–8.3) (*P* = 0.76) for isoniazid. Age, gender or extrapulmonary TB also did not predict a low concentration in bivariate analysis. Patient weight was not available in all patients for stratification by mg/kg dosing. Fifteen patients had follow-up concentrations after dose adjustment and 12 (80%) increased to the expected range (including all for rifampin). No complications or major toxicity from dose adjustment were reported.

Of 16 diabetic patients with pulmonary TB, 14 (88%) converted their sputum culture to negative in <2 months, including 9 of 11 (82%) patients for whom either rifampin or isoniazid was dose increased. There were no deaths reported over a median follow-up time of 10.5 months (IQR 8–12.25 months). The two diabetics that failed to culture convert in two months were both foreign born males of advanced age (88 and 71 years); one of whom had low *C*_2 hr_ isoniazid and rifampin where each medication was corrected to the expected range, and the other with low *C*_2 hr_ rifampin in whom a repeat concentration was not repeated after dose increase. Including the diabetics with pulmonary TB that failed to culture convert in <2 months or those with extrapulmonary TB that were later deemed to have slow response, the mean number of slow responders was 1.2 per month (12.5% diabetic) during the early TDM period, decreased from preintervention rates of 1.6 per month (40% diabetic) [7].

## 4. Discussion

A statewide initiative of early TDM in diabetics starting anti-TB therapy found that the majority have a low serum *C*_2 hr_ value of isoniazid or rifampin that corrected to the expected range with a single dose increase. The process was operationally feasible and accepted by health departments with capture of more than 80% of all diabetics treated for active TB. The target of performing early TDM at 2 weeks after treatment initiation was closely approximated in most diabetics.

To our knowledge, this is the first programmatic initiative to correct low isoniazid and rifampin concentrations routinely in all diabetics. The few observational studies that have examined anti-TB pharmacokinetics specifically in diabetics did not include dose correction and focused on rifampin. For example, rifampin exposure, as measured by sampling of serum throughout the daily dosing interval, was 2-fold lower in diabetic patients compared to age and gender matched controls from Indonesia when performed during the continuation phase of treatment [13]. However, these findings were not reproduced when studied in a similar cohort of subjects during the first two weeks of therapy, but the comparator group also had a high proportion with low rifampin exposure and the authors suggest that patient's weight and hepatic induction may be more contributory to the lower rifampin concentrations they had previously observed during the later stage of the treatment course [14]. Similarly, a recent study of *C*_2 hr_ and *C*_6 hr_ concentrations of rifampin from Peru found that the majority of diabetics have low rifampin concentrations though not significantly different than non-diabetics, but nearly 85% of the total population studied had peak concentrations below the expected range, including a notable proportion with undetectable *C*_2 hr_ values, which may have made differences in the diabetic and non-diabetic populations difficult to detect [15]. Furthermore, rifampin solubility is affected by gastric pH and transit time, conditions which are influenced by chronic hyperglycemia and can fluctuate significantly within an individual patient [16, 17]. Thus, while the programmatic initiative of checking only a *C*_2 hr_ concentration may miss a proportion of those with a delayed peak concentration, the operational decision to test for both isoniazid and rifampin at single time point was found most feasible in our setting. Additionally, the lack of significant side effects with a single dose correction of either medication suggests that a delayed time to peak, if present, was not clinically significant.

Therefore, given the high frequency of low anti-TB drug concentrations in diabetics, a randomized trial of TDM with dose correction may be the best means of quantifying the contribution of pharmacokinetic optimization to both early and late markers of treatment outcome. For instance, a low rifampin concentration may not be sufficient to affect late markers such as cure or relapse in a subject with a highly rifampin susceptible *M. tuberculosis* isolate or adequate concentrations of the other anti-TB drugs in the regimen. However, dose correction to the higher range of expected peak concentrations may hasten the early treatment response [18]. This may be particularly important for subjects with *M. tuberculosis* isolates with higher minimum inhibitory concentrations still considered susceptible by conventional testing [8]. The early TDM initiative found fewer diabetics with low rifampin concentrations compared to our prior study in Virginia when TDM was restricted to only those patients with slow response, and low isoniazid and rifampin *C*_2 hr_ concentrations were found in 63% and 76% of diabetics, respectively [7]. We speculate this may be an indication of the relative importance of rifampin in the rapidity of treatment response for diabetics in our setting. Indeed, recent attention to the optimization of rifampin concentrations demonstrates significantly improved bactericidal activity with dose increase as measured by sputum colony counts, and promising clinical trials are underway to study higher dose rifampin for shortening treatment duration [18–20]. Therefore, following the outcomes of these trials, diabetics may be an ideal subpopulation in which consider a higher initial dose of rifampin.

A high proportion of diabetics with early TDM had a favorable time to sputum culture conversion of <2 months. The time to culture conversion has been modestly delayed in other studies of diabetics when compared to non-diabetic controls likely related to a higher bacillary burden at presentation [5, 21, 22]. While our current study did not permit comparison of time to culture conversion in TB cases for whom TDM was not performed, the decreased total number of slow responders during the period of the early TDM initiative and the decreased proportion of diabetics that developed slow response compared to historical norms provide support that early correction of low drug concentrations may avert slow response in some diabetics. This finding is of considerable programmatic value as the total treatment duration depends upon the microbiological and symptomatic response in the first two months of therapy. Given current financial constraints placed on state TB control programs, avoiding an extended duration of directly observed therapy can be resource sparing and cost saving. 

Diabetes is currently identified by patient self-report or chart review by the state TB control program. Yet in studies of active screening by fasting blood glucose or HbA1c in new TB patients without a known history of diabetes, the rate of identification of new cases of diabetes ranged from 2 to 35% depending on the population of study [23]. In the Indian state of Kerala for instance, only 4 new TB cases were needed to be screened by HbA1c in order to identify 1 new case of diabetes [24]. Thus, the burden of diabetes among slow responders to anti-TB treatment may be underestimated in our setting. Active screening for diabetes in all new TB patients may identify a subset of patients otherwise eligible for early TDM. Consequently, plans to start active screening for diabetes with HbA1c are now underway in Virginia.

There are several limitations to this study given the necessity for retrospective study of an initiative in place for all diabetics and the inability to randomize diabetics to early TDM or the prior standard of care. While a drop in the total number of slow responders compared to preinitiative rates was observed, nurse case managers or TB clinicians may have possessed an unintended bias and preferentially failed to identify a diabetic patient as slow to respond once early TDM had been performed. If occurring, however, the bias would largely be limited to patients without the objective finding of sputum culture conversion. In addition, a minority of eligible diabetics did not have early TDM performed, and while they were not later identified as having slow response, the reasons for lack of the implementation of the initiative in these patients were not known. 

Furthermore, sputum culture conversion and identification of slow response were used as proxy for predicting total treatment duration and the intensity of programmatic resources required in management. Thus, further cost-effectiveness analysis would require long-term followup and comprehensive comparison of data from matched non-diabetic controls that were not currently available. Lastly, while insulin use did not further risk stratify for low drug concentrations among diabetics, little else was known about diabetic disease severity. Furthermore, details of comorbid medical conditions, patient weight, or medication use may have additionally refined the interpretation of low drug concentrations or markers of treatment response such as sputum culture conversion. 

## 5. Conclusions

In summary, early TDM in diabetics starting anti-TB therapy revealed that the majority had a low isoniazid or rifampin serum concentration corrected to the expected range with a single dose increase and no major reported toxicity. Diabetics with early TDM and pulmonary TB had a favorable time to sputum culture conversion and the total statewide burden of slow response appeared to be minimized during the period of the initiative. Thus, early TDM for diabetics can be considered in settings of high diabetes/TB coprevalence where slow response and prolonged treatment duration are programmatic concerns.

## Figures and Tables

**Table 1:** Baseline characteristics of adults with drug-susceptible tuberculosis referred for therapeutic drug monitoring (TDM) based on slow response or early TDM if diabetic.

Characteristic	Diabetes (early TDM) N = 21	Slow response (standard TDM) N = 14	P value
Age, mean years ± SD	57 ± 17	46 ± 12	*P* = 0.04
Gender, male (%*N*)	15 (71)	11 (79)	*P* = 0.69
Prior episode of TB, *n* (%*N*)	0	2 (14)	*P* = 0.17
Pulmonary TB only, *n* (%*N*)	17 (81)	8 (57)	*P* = 0.65
Foreign born (%*N* with confirmed status*)	15 (79)	12 (92)	*P* = 0.63
HIV infected (%*N* with confirmed status*)	0	1 (11)	*P* = 0.43
Insulin dependence, *n* (%*N*)	10 (48)	N/A	N/A
Days to TDM from treatment initiation, median days (IQR)	23 ± 16	88 ± 54	*P* = 0.003

Slow response patients did not include diabetics (see Figure 1).*Missing values include foreign born status in 2 diabetics and 1 patient with slow response and HIV status in 4 patients with slow response. N/A: not applicable.

**Table 2:** Distribution of estimated peak concentrations (*C*_2 hr_) for isoniazid and rifampin.

Drug	Diabetics (early TDM)	Nondiabetic slow responders (standard TDM)	*P* value
*Rifampin* (expected range 8–24 *μ*g/mL)	*N* = 20	*N* = 14	
Mean *C*_2 hr_ *μ*g/mL ± SD	6.6 ± 4.3	8.2 ± 6.2	*P* = 0.40
Below expected range (%*N*)	12 (60)	4 (41)	*P* = 0.31

*Isoniazid (daily)* (expected range 3–6 *μ*g/mL)	*N* = 17	*N* = 8	
Mean *C*_2 hr_ *μ*g/mL ± SD	2.1 ± 1.5	3.1 ± 1.1	*P* = 0.11
Below expected range (%*N*)	11 (65)	5 (63)	*P* = 0.92

*Isoniazid (intermittent) * ** **(expected range 9–18 *μ*g/mL)	*N* = 4	*N* = 6	
Mean *C*_2 hr_ *μ*g/mL ± SD	6.0 ± 3.0	11.3 ± 2.5	*P* = 0.03
Below expected range (%*N*)	3 (75)	1 (17)	*P* = 0.19

TDM: therapeutic drug monitoring (see Figure 1).
